# The effect of tai chi intervention on NLRP3 and its related antiviral inflammatory factors in the serum of patients with pre-diabetes

**DOI:** 10.3389/fimmu.2022.1026509

**Published:** 2022-09-28

**Authors:** Shujuan Hu, Yingxing Hu, Peilin Long, Peixiong Li, Ping Chen, Xianwang Wang

**Affiliations:** ^1^ School of Education and Physical Education, Yangtze University, Jingzhou, China; ^2^ School of Physical Education and Science, Jishou University, Jishou, China; ^3^ Department of Biochemistry and Molecular Biology, Center for Molecular Medicine, Health Science Center, Yangtze University, Jingzhou, China

**Keywords:** tai chi intervention, NLRP3 inflammasome, pre-diabetes mellitus, inflammatory factors, insulin resistance

## Abstract

**Background:**

NLRP3 inflammasome and its related antiviral inflammatory factors have been implicated in the pathogenesis of type 2 diabetes mellitus (T2DM) and insulin resistance, but its contribution to pre-diabetes remains poorly understood.

**Objective:**

To investigate the effects and the potential mechanism of Tai Chi intervention on NLRP3 inflammasome and its related inflammatory factors in the serum of middle-aged and older people with pre-diabetes mellitus (PDM).

**Methods:**

40 pre-diabetic subjects were divided into a pre-diabetic control group (PDM-C group, N=20) and a Tai Chi group (PDM-TC group, N=20) by random number table. 10 normoglycemic subjects (NG) were selected as controls. We measured clinical metabolic parameters and collected blood samples before and after the 12 weeks of Tai Chi intervention. Antiviral inflammatory factors in serum were detected by enzyme-linked immunosorbent assay.

**Results:**

The blood glucose, insulin resistance, and inflammation in PDM groups were higher than those in the NG group (*P*<0.05 and *P*<0.01, respectively). The results also suggested that 12 weeks of Tai Chi intervention could reduce body weight, blood pressure, blood glucose, insulin resistance, blood lipid, and the expressions of serum inflammatory factors in the pre-diabetic population.

**Conclusion:**

Tai Chi intervention may improve blood glucose, lipid levels, and insulin resistance in middle-aged and elderly pre-diabetic patients by reducing the level of NLRP3 inflammasome and its related inflammatory factors.

## Introduction

Diabetes mellitus is a common and complex chronic disease that has become a serious threat to human health after cancer, cardiovascular, and cerebrovascular diseases, of which 90% are type 2 diabetes mellitus (T2DM) (1). Diabetes mellitus (in particular T2DM) develops slowly and may have a pre-diabetes mellitus (PDM) state, which is a high-risk state for diabetes (2). PDM is the only stage that can be reversed the occurrence of diabetes as it is a necessary stage for the normal progression to T2DM (2). Therefore, it is a wise option to ameliorate diabetes by developing an effective strategy for the PDM population. The Guidelines for Prevention and Treatment of T2DM in China (2020 edition) explicitly recommended that the prevention and relief of T2DM require adjustment of unhealthy lifestyle and persistence of exercise (1). Naturally, exercise is an important scheme of lifestyle adjustment, which has been recommended for managing pre-diabetes and diabetes. Previous studies have found that exercise intervention significantly improved fasting blood glucose (FBG), plasma glucose after 2 hours (2 hPG), and Glycosylated hemoglobin (HbA_1_C) in patients with PDM (3). In addition, exercise intervention has been shown to ameliorate glucose tolerance, and effectively prevent impaired glucose tolerance from progressing to diabetes (4).

Inflammation is an adaptive biological response of the immune system (5). Chronic inflammation is an important pathophysiological factor leading to diabetes (6), which is manifested by higher levels of Nod-like receptor protein 3 (NLRP3), Caspase-1, Interleukin-1β (IL-1β), and various antiviral inflammatory cytokines (7), inducing a strong inflammatory response in the body. NLRP3 inflammasome is a multi-protein complex composed of the nod-like receptor (NLR) family core member (NLRP3), apoptosis-associated spot-like protein (ASC), and Caspase-1 (8). Nuclear factor κB (NF-κB), and reactive oxygen species (ROS) have been regarded as the important upstream signal to activate the NLRP3 inflammasome (9). Activation of NLRP3 inflammasome could activate Caspase-1, which cleaves the pro-IL-1β precursor to form mature IL-1β secreted out of the cell, thereby inducing the body’s inflammatory response (10). In addition, the NLRP3 inflammasome has become a regulator of inflammatory response and protective immunity (11), which plays an important role in the antiviral innate immune signaling pathway (12).

Much evidence has shown that aerobic exercise could reduce the expressions of NLRP3, Caspase-1, IL-1β and other inflammatory factors (13, 14). Zaidi et al. found that one year of exercise training in patients with T2DM significantly reduced the levels of pro-inflammatory markers, especially IL-18 (15). After 8 weeks of aerobic exercise, the activity of NF-κB and NLRP3 in the prefrontal cortex of diabetic rats decreased. The activity of Phosphatidylinositol 3-hydroxy kinase (PI3K)/protein kinase B (Akt) was enhanced, and the insulin signaling pathway was improved by inhibiting the inflammatory signaling (14). These findings suggested that moderate-intensity aerobic exercise may ameliorate insulin sensitivity by inhibiting over-activation of the NLRP3 inflammasome, thereby alleviating insulin resistance. In brief, aerobic exercise could relieve diabetes by suppressing inflammation.

Tai Chi is an aerobic exercise with a long history, and Tai Chi has been widely used in the clinical prevention and treatment of diabetes. At the same time, the exercise intensity is moderate, which is favored by the middle-aged and elderly. Tai Chi has been shown to improve the level of blood glucose and lipid in diabetic patients, and the potential mechanism may be driven by insulin resistance, and reduction in inflammatory factors (16, 17). Studies have found that Tai Chi can stimulate innate and adaptive immune cell responses and regulate inflammatory biomarkers, enhancing participants’ immune system function (5, 18). However, whether Tai Chi plays a vital role in regulating pre-diabetic symptoms remains unknown. The evidence of beneficial therapeutic effects of exercise interventions on reducing inflammatory markers is also unclear in pre-diabetic rat models (19) and the pre-diabetic population. Therefore, we aimed to explore the effect and potential mechanism of Tai Chi intervention on the NLRP3 inflammasome and its related inflammatory factors in pre-diabetic population, and we seek an economical and effective strategy to alleviate and improve diabetes.

## Materials and methods

### Studied subject and study design

In this study, a randomized controlled study design was adopted, cases were screened strictly following the inclusion and exclusion criteria established in the study protocol, eligible cases were randomly grouped, intervened, observed and followed up, and relevant data were collected. The sample size was determined based on literature reports of similar studies (20). A total of 40 pre-diabetic patients and 10 healthy subjects recruited from Jishou University from April to July 2021 were selected, among which 2 patients withdrew due to the reason of health. At last, 38 participants were randomly divided into the PDM control group (PDM-C, N=19) with an age of (61.58 ± 6.62) years and a height of (1.60 ± 0.09) meters; the PDM Tai Chi group (PDM-TC, N=19), age was (62.68 ± 7.33) years, height was (1.57 ± 0.06) meters; normoglycemic subjects (NG, N=10), age of (55.20 ± 7.45) years, the height of (1.59 ± 0.07) meters. All groups had no significant differences in age, height, and body weight (P>0.05).

Eating habits of all recruited participants are relatively stable. They were asked to continue their daily routines without changing physical activity and eating habits. Monthly one-on-one interviews with a valid questionnaire, including nutritional intake and physical activity, were conducted to assess their compliance. All participants underwent clinical assessment at recruitment to the project, followed by an oral glucose tolerance test (OGTT) and blood test. All tests were performed before the intervention and repeated 3 months following each participant’s final exercise session. The Ethics Committee approved this study of Jishou University (approval number: JSDX-2021-0055). All study participants provided written informed consent.

Inclusion criteria: 1. PDM patients (N=38), (i) Subjects with impaired fasting glucose (IFG) or impaired glucose tolerance (IGT), defined as FBG 100-125 mg/dL or 2 hPG 140-199 mg/dL. (ii)Age: 50-70 years old. (iii) Ability to perform exercise training. 2. NG group (N=10), (i) Subjects with normal fasting glucose and normal glucose tolerance, defined as FBG <100 mg/dL and 2 hPG <140 mg/dL. (ii)Age: 45-70 years old. Exclusion criteria: (i) Highly active lifestyle. (ii) Patients with type 1 or 2 diabetes, other special types of diabetes and abnormal liver and kidney function were excluded. (iii) Patients with a history of cancer and other serious diseases were excluded.

### Tai Chi exercise intervention

#### PDM-TC group

All patients underwent a 12-week Tai Chi intervention. 24 simplified Tai Chi intervention was conducted for 12 weeks under the guidance of a professional Tai Chi instructor (group instruction). Practice venue: Jishou University New Campus sports ground. Practice period: April to July 2021. The training frequency was four sessions per week for 12 weeks, for a total of 48 pieces of training. Only subjects who performed at least 80% of all planned training were included in this study. Each training lasted approximately 80 min, beginning with a warm-up (20 min), Tai Chi exercises (50 min), followed by relaxation exercises (10 min). The first 1-3 weeks of Tai Chi learning period, 4-12 weeks of Tai Chi consolidation and strengthening.

Before enrollment, the researcher conducted relevant training and education for all PDM-TC group subjects, and informed them of the specific details of the exercise program and matters needing attention. The blood pressure and heart rate of subjects were measured before each exercise. Exercise intensity was maintained to keep the heart rate within 50%~60% of the maximum heart rate (male maximum heart rate=220-age, female maximum heart rate=210-age), and wear a polar watch randomly for real-time monitoring. The most significant feature of the exercise intervention is that it was 100% supervised to ensure uniformity among patients in the exercise intervention. If the patients show any discomfort, the exercise will be terminated immediately.

### PDM-C group and NG group

Do not exercise regularly in any other way than to maintain their previous lifestyle. Both groups were visited weekly to know their states of life, to ensure they did not engage in other forms of disciplined exercise and did not change their diet.

### Biochemical measurements of subjects

#### Anthropometrics and body composition

Anthropometric measurements and body composition analyses were performed in the fasted state using a calibrated body composition analyzer Model N40 (Korea). BMI was calculated as body mass (kg) divided by height (m) squared. Blood pressure is measured continuously 3 times in a calm state, and its average value is taken. A tape measure was used to measure the subjects’ waist and hip measurements, and the waist-hip ratio was calculated.

#### OGTT and laboratory measurements

The OGTT procedure was conducted following ADA recommendations (21). Blood glucose was measured in plasma using a blood glucose detector with Kyoto U-Test (Kyoto, Japan). Blood samples were collected from all subjects before and after Tai Chi intervention. Fasting for more than 8 h was required, and 5 ml of venous blood was taken on an empty stomach from 7:00 to 9:00 the next morning. Fasting insulin (FINs), total cholesterol (TC), triglyceride (TG), low-density cholesterol (LDL-C), high-density cholesterol (HDL-C), FBG, and other biochemical indexes were determined by Hitachi 7600 automatic biochemical analyzer. HbA_l_C was determined by Hitachi 7170A automatic glycated hemoglobin analyzer. The homeostatic model assessment for insulin resistance was calculated to evaluate insulin resistance: homeostatic model assessment for insulin resistance (HOMA-IR) = (FBG×FINs)/22.5. The concentrations of NF-κB, ROS, NLRP3, ASC, Caspase-1, GSDMD, IL-1β, and IL-18 in serum of all subjects were detected by enzyme-linked immunosorbent assay (ELISA). The ELISA kits were purchased from Sin-Troch (China). The instrument was Rayto and RT-6100 microplate reader, and the operation was completed according to the kit instructions. The operation is completed according to the operating instructions.

### Statistical analyses

SPSS23.0 statistical software was used to process the measured data, and the experimental data were expressed as the mean ± standard deviation (x̄ ± s). Paired *t*- test was used for intra-group comparison before and after the intervention. Comparison among the three groups was performed by one-way ANOVA. *P*<0.05 or *P*<0.01 means the difference is statistically significant.

## Results

### Tai Chi intervention reduced the related indexes of body weight and blood pressure in patients with pre-diabetes

To determine the effect of Tai Chi intervention on weight and blood pressure indexes in the pre-diabetic population. As shown in **Table 1**, of 48 enrolled subjects, 10 (21%) were normoglycemic; 38 (79%) were pre-diabetic with impaired fasting glucose or glucose tolerance. PDM participants were randomly divided into the PDM-C group (N=19) and the PDM-TC group (N=19). 10 NG group (N=10) were selected as controls. The results showed no statistically significant difference in Wt, BMI, SBP, DBP and WHR among PDM-C, PDM-TC, and NG groups (*P*>0.05). After 12 weeks of Tai Chi intervention in the PDM-TC group, there were significant differences in Wt, BMI, SBP, and DBP (*P*<0.01). Compared with the PDM-C group, we found that there were significantly decreased DBP and WHR in the PDM-TC group (*P*<0.01). These results demonstrated that Tai Chi intervention for 12 weeks could reduce body weight and blood pressure in the pre-diabetic population.

**Table 1:** Clinical characteristics of subjects.

Parameter (Unit)	PDM-TC (N = 19)		PDM-C (N = 19)		NG (N = 10)	
	Before	After	Before	After	Before	After
Wt (kg)	61.58 ± 9.42	59.21 ± 9.05^b^	65.49 ± 11.08	65.37 ± 11.05	60.45 ± 7.17	60.38 ± 8.27
BMI (kg/m^2^)	24.99 ± 3.64	24.15 ± 3.42^b^	25.41 ± 2.57	25.34 ± 2.38	23.89 ± 1.80	23.83 ± 1.97
SBP (mmHg)	143.05 ± 19.80	124.79 ± 14.56^b^	138.16 ± 18.37	136.53 ± 19.66	128.20 ± 14.73	126.90 ± 16.70
DBP (mmHg)	80.84 ± 8.20	74.00 ± 10.01^bd^	84.32 ± 7.82	82.27 ± 7.04	84.80 ± 13.61	83.10 ± 10.50
WHR	0.91 ± 0.05	0.89 ± 0.04^d^	0.91 ± 0.05	0.96 ± 0.04	0.89 ± 0.06	0.91 ± 0.06
FBG (mmol/L)	6.39 ± 0.31^d^	6.04 ± 0.31^bd^	6.40 ± 0.31	6.60 ± 0.34	5.42 ± 0.42	5.44 ± 0.30
2 hPG (mmol/L)	9.17 ± 2.17^d^	7.56 ± 2.47^bc^	8.62 ± 0.77	8.66 ± 0.72	6.70 ± 0.73	6.94 ± 0.72
CRP (mg/dl)	1.94 ± 0.11^c^	1.86 ± 0.11^bd^	2.06 ± 0.15	2.09 ± 0.13	1.95 ± 0.12	1.92 ± 0.11
HbA_1_C (%)	6.23 ± 0.33^d^	6.00 ± 0.34^bd^	6.24 ± 0.29	6.32 ± 0.50	5.60 ± 0.28	5.65 ± 0.18
HbA_1_ (%)	7.65 ± 0.53^d^	7.39 ± 0.44^bd^	7.65 ± 0.39	7.56 ± 0.65	6.64 ± 0.44	6.62 ± 0.36
FINs (μU/mL)	12.22 ± 5.97^c^	9.73 ± 4.72^bc^	13.98 ± 5.23	13.08 ± 4.62	8.76 ± 2.84	9.54 ± 3.28
HOMA-IR	3.13 ± 1.71^d^	2.27 ± 1.15^bd^	3.74 ± 1.41	3.66 ± 1.35	1.92 ± 0.73	2.04 ± 0.81
TG (mmol/L)	1.65 ± 0.83	1.56 ± 0.86	2.00 ± 0.87	2.19 ± 0.95	1.44 ± 1.07	1.68 ± 0.73
TC (mmol/L)	5.58 ± 1.05	5.33 ± 1.05^b^	5.03 ± 1.01	5.06 ± 0.92	5.17 ± 1.05	5.16 ± 0.78
HDL-C (mmol/L)	1.41 ± 0.34	1.41 ± 0.33	1.58 ± 0.52	1.65 ± 0.63	1.37 ± 0.32	1.34 ± 0.28
LDL-C (mmol/L)	3.32 ± 0.84	3.11 ± 0.88^b^	3.42 ± 0.72	3.44 ± 0.73	3.15 ± 0.67	3.17 ± 0.49

Comparison in the group,^a^P < 0.05, ^b^P < 0.01; Comparison between groups, ^c^P < 0.05, ^d^P < 0.01. Wt, weight; BMI, body mass index; WHR, waist-to-hip ratio; FBG, fasting blood glucose; 2 h PG, 2 hours plasma glucose; HDL-C, high-density lipoprotein cholesterol; LDL-C, low-density lipoprotein cholesterol; TG, triglycerides; TC, total cholesterol; CRP, C-reactive protein; HbA_1_c, haemoglobinA1c; HbA_1_, total glycosylated hemoglobin; FINs, fasting insulin; HOMA-IR, homeostatic model assessment for insulin resistance.

### Tai Chi intervention decreased blood glucose and insulin resistance in pre-diabetes

To examine whether Tai Chi intervention for 12 weeks influences blood glucose and insulin resistance in pre-diabetes, patients’ blood analysis was performed. As shown in **Table 1**, before 12 weeks of Tai Chi intervention, we observed that there are significant differences in FBG, 2 hPG, HbA_1_C, HbA_1_, FINs, and HOMA-IR among PDM-C, PDM-TC, and NG groups (*P*<0.05 or *P*<0.01), suggesting that the blood glucose and insulin resistance in PDM groups were significantly higher than those in NG group. Nevertheless, there were no significant differences between the PDM-C and PDM-TC groups in FBG, 2 hPG, HbA_1_C, HbA_1_, FINs, and HOMA-IR (*P*>0.05). After 12 weeks of Tai Chi intervention in the PDM-TC group, FBG, 2 hPG, HbA_1_C, HbA_1_, FINs, and HOMA-IR were decreased. Compared with the PDM-C group, we found that FBG, 2 hPG, HbA_1_C, and HbA_1_ were reduced significantly in the PDM-TC group (*P*<0.05 or *P*<0.01). Specifically, there was statistical significance in insulin resistance-related indexes, including FINs and HOMA-IR (*P*<0.05 or *P*<0.01) between the PDM-C and PDM-TC groups. Taken together, 12 weeks of Tai Chi intervention could decrease blood glucose and insulin resistance in pre-diabetes (**Table 1**).

### Tai Chi intervention has a positive effect on blood lipid in pre-diabetes

To detect the influence of Tai Chi intervention on blood lipid in the pre-diabetic population. Before Tai Chi intervention, there were no significant differences among the PDM-C group, PDM-TC group, and NG group in blood lipid-related indexes, including TG, TC, HDL-C, and LDL-C (*P*>0.05), respectively, indicating that the difference is not obvious in blood lipids among the three groups. After 12 weeks of Tai Chi intervention in the PDM-TC group, there were significant differences in TC and LDL-C (*P*<0.01), however, the improvements in TG and HDL-C were not significant (**Table 1**). These results indicated that a 12-week Tai Chi intervention positively affects blood lipid-related indexes in the pre-diabetic population. Compared with the PDM-C group, we found no significant difference in TG, TC, HDL-C, and LDL-C (*P*>0.05), possible resulting from the small sample size and the short intervention time.

### Tai Chi intervention reduced serum inflammatory factors in prediabetes

**Figure 1:**
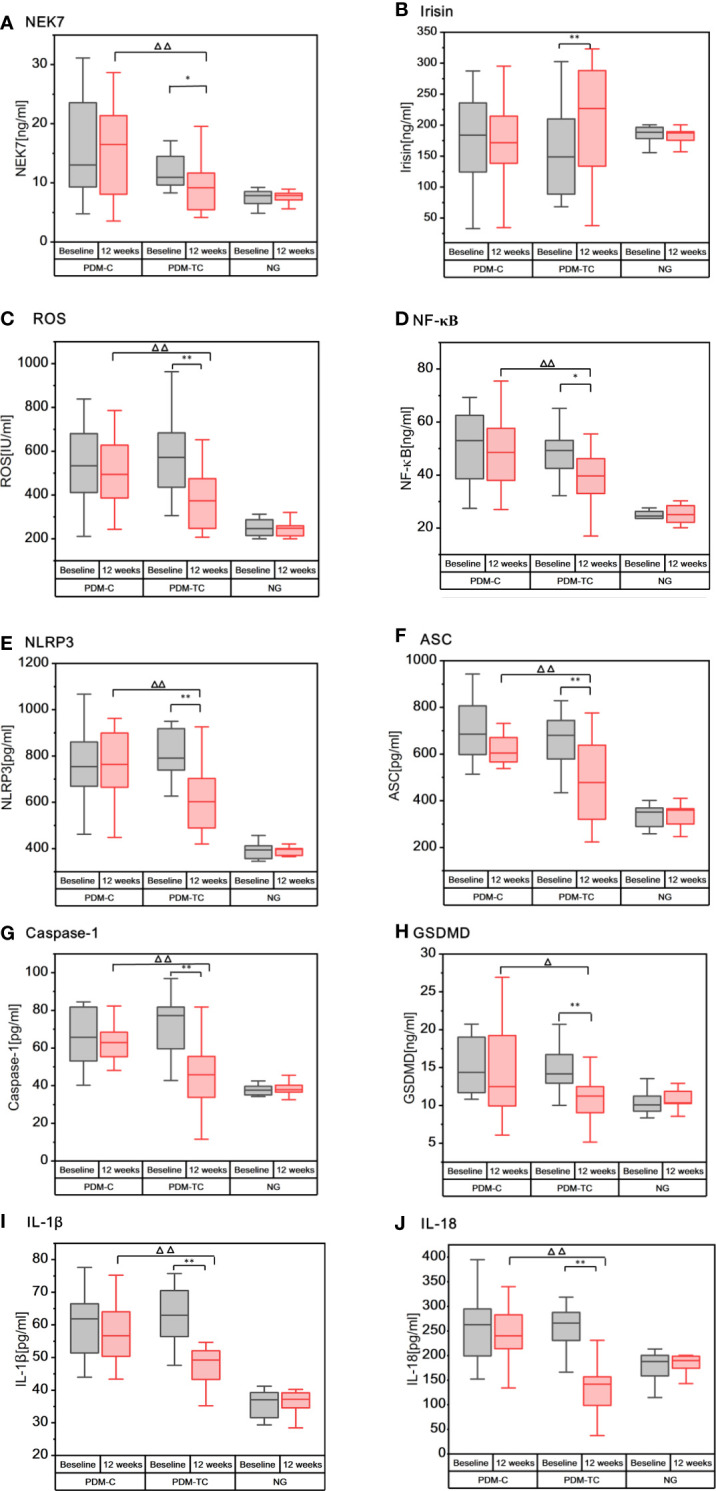
The expressions of inflammatory cytokines in the serum of patients with PDM-C, PDM-TC and NG groups, before and after 12 weeks of Tai Chi intervention. “*” indicates that there are differences within the group (*P* < 0.05), and “**” indicates that there is significant difference in the group (*P* < 0.01). “Δ“ indicates differences between the PDM-TC group and the PDM-C group (*P* < 0.05), and “ΔΔ“ indicates significant differences between the PDM-TC group and the PDM-C group (*P* < 0.01).

An ELISA assay was employed to confirm the effect of Tai Chi intervention on serum inflammatory factors in the pre-diabetic population. Before Tai Chi intervention, we did not find any significant differences in inflammation markers in the serum between PDM-TC and PDM-C groups. As shown in **Figures 1A–J**, most inflammatory indexes in the NG group were lower than those in the PDM groups. However, the mean value of irisin was slightly higher in the NG group than in the PDM groups. Intra group comparison results showed that there were significantly reduced the expressions of NEK7, ROS, NF-κB, NLRP3, ASC, Caspase-1, GSDMD, IL-1β, and IL-18, while the expression of irisin was increased in PDM-TC group after 12 weeks Tai Chi intervention (*P*<0.05 or *P*<0.01). Compared with the PDM-C group, the expressions of NEK7, ROS, NF-κB, NLRP3, ASC, Caspase-1, IL-1β, and IL-18 were significantly decreased in the PDM-TC group (*P*<0.05 or *P*<0.01). However, there was no significant difference in the expression level of irisin in the PDM-TC group. These results suggest that Tai Chi intervention for 12 weeks can significantly reduce the expression of serum inflammatory factors in pre-diabetes.

## Discussion

Diabetes is a metabolic disease mainly characterized by hyperglycemia, presenting a “chronic low-grade inflammatory” state (22). Chronic inflammation is also considered a key inducer in the development of diabetes and permeates the whole process of diabetes (23). NLRP3 inflammasome, IL-1β, and IL-18 can affect blood glucose control and insulin resistance, which are related to the pathogenesis of diabetes (23, 24), and play an important role in diabetes-induced systemic chronic inflammation and insulin signal transduction (15, 25). Our study showed that the expressions of NLRP3 inflammasome and other antiviral inflammatory cytokines in the NG group were lower than those in the PDM groups, suggesting that the pre-diabetes is indeed in a chronic inflammatory state. Consistent with our findings, studies have found that NLRP3 inflammasome, Caspase-1, IL-1β, IL-18, and other inflammatory factors were significantly increased in multiple tissues of diabetic patients (26, 27). Many studies showed that hyperglycemia could produce excessive ROS (28) and activate NF-κB (29), thereby triggering the activation of intracellular signal transduction and NLRP3 inflammasome in the occurrence and development of diabetes (30). It is reported that NEK7 directly regulates the activation of NLRP3 inflammasome (31). A previous study also showed that the expressions of NEK7 and NLRP3 inflammasome in vascular cells of patients with diabetes were significantly increased (32). In this work, we found that ROS, NF-κB, and NEK7 were significantly higher in PDM groups than in the NG group (as shown in **Figures 1A, C, D** **)**. Additionally, irisin plays a crucial role in diabetes and energy metabolism (33). A previous study showed that the level of circulating irisin in patients with diabetes was lower when compared with that of the non-diabetic control group (34). Our study found that the average irisin of the NG group was slightly higher than that of the PDM groups (as shown in **Figure 1B**), but there was no statistical significance. The possible reasons are that the sample size is small and the study object is pre-diabetes. In addition, irisin can reduce the excessive production of ROS and oxidative stress (35), and inhibit the formation and activation of NLRP3 inflammasome (36). To sum up, ROS, NF-κB, and NEK7 could activate NLRP3 inflammasome, while irisin inhibits the activation of these inflammatory cytokines.

Aerobic exercise is believed to be a promising intervention to reduce the expressions of ROS, NF-κB (37), NEK7, NLRP3, ASC, Caspase-1, IL-1β, and other inflammatory factors (38–40), and improves diabetes-induced inflammation and reduces insulin resistance. Tai Chi is a typical aerobic exercise, as well as a physical and mental exercise method of both internal and external cultivation and coordinating the balance of mind-body (41). The data detected by ELISA suggested that Tai Chi intervention for 12 weeks could significantly reduce the concentration of the serum inflammatory factors including ROS, NF-κB, NEK7, NLRP3, ASC, Caspase-1, GSDMD, IL-1β, and IL-18 in patients with pre-diabetes, indicating that Tai Chi intervention could relieve vascular and systemic inflammation. Another important finding of our study is that the level of irisin in the blood of patients with pre-diabetes increased slightly after 12 weeks of Tai Chi intervention (P<0.01) (as shown in **Figure 1B**), which is consistent with the study of Jia (42). Therefore, 12 weeks of Tai Chi intervention can significantly decrease the expressions of NEK7, NLRP3, ASC, Caspase-1, GSDMD, IL-1β, and IL-18, while increasing the expression of irisin in pre-diabetes.

We showed that the 12 weeks of Tai Chi intervention effectively alleviated glucose homeostasis and anthropometric parameters in both PDM-TC and PDM-C relative to normoglycemia. Thus, Tai Chi is an important form of exercise in pre-diabetes, which may be useful in preventing the development of T2DM. Previous studies have also confirmed that Tai Chi intervention can significantly reduce TC,TG, FBG, and HbA_1_C in T2DM patients (43). This study also found that Tai Chi intervention can reduce the expressions of blood glucose, blood lipid, and inflammatory factors in pre-diabetes. However, does Tai Chi intervention improve blood glucose, blood lipid, and insulin resistance by reducing the expression of inflammatory factors?

**Figure 2:**
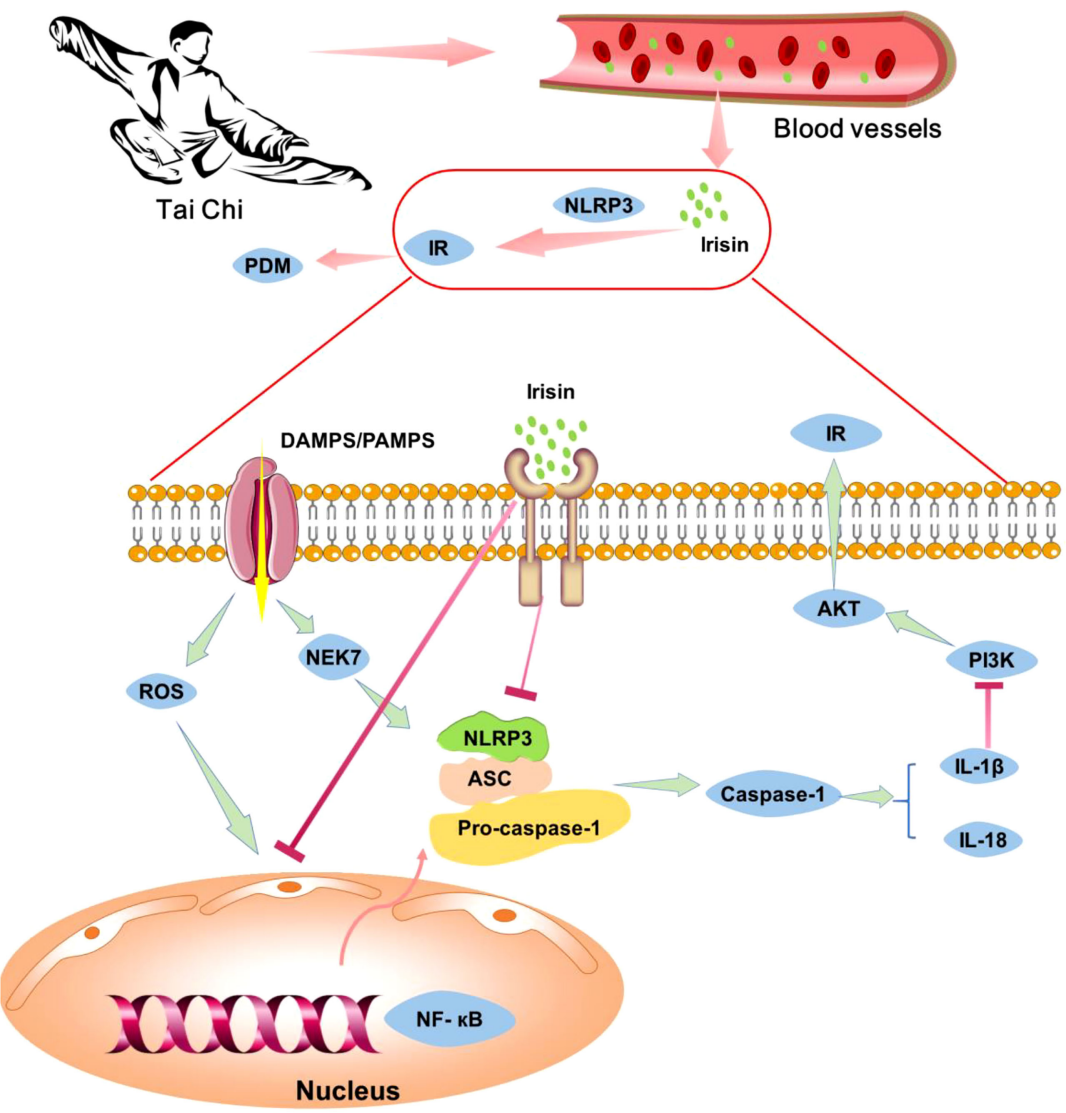
The potential mechanism of Tai Chi intervention relieves pre-diabetes by inhibiting inflammation cytokines. Tai Chi intervention can increase the level of irisin in the blood, thus inhibiting the expression of NLRP3 inflammasome and other inflammatory factors, enhancing insulin receptor sensitivity, relieving insulin resistance and pre-diabetes.

Previous studies have shown that ROS, NF-κB, and NEK7 can activate the NLRP3 inflammasome, which secretes IL-1β. IL-1β activates C-Jun N-terminal kinase (JNK) and induces serine phosphorylation of insulin receptor substrate 1 (IRS-1), inhibits the expression of Akt protein kinase (44) and weakens the insulin/PI3K/Akt signaling pathway in insulin-sensitive tissues, leading to insulin resistance (45). Irisin can inhibit the formation and activation of NLRP3 inflammasome (36), thereby improving insulin resistance. Studies have shown that exercise can increase the expression of irisin in skeletal muscle, increasing of circulating irisin (35). Therefore, we speculated that Tai Chi intervention might increase irisin level in skeletal muscle, lead to a high concentration of irisin in blood, and thus inhibit the expressions of NLRP3 and other inflammatory factors. The decrease of inflammatory factors could further enhance insulin receptor sensitivity and improve insulin resistance (**Figure 2**). In general, Tai Chi intervention may improve insulin resistance by reducing the expression of inflammatory factors.

However, as the objects of study are the pre-diabetes population, the expressions of inflammatory-related factors in the tissues cannot be detected. It is suggested that the mouse model should also be used in future studies. Secondly, the sample size can be increased in the follow-up study. Finally, its follow-up research can try applying inflammation-related factors to the study of exercise in mice with pre-diabetes to explore the molecular mechanism of how different exercise patterns reduce inflammation.

## Conclusion

Tai Chi intervention can reduce blood glucose, blood lipid, and insulin resistance levels and decrease the serum levels of inflammatory factors, including NF-κB, ROS, NLRP3, IL-1β, and IL-18 in pre-diabetes. The potential mechanism is that Tai Chi intervention could increase the level of irisin in the blood, and inhibit the expression of the NLRP3 inflammatory signal pathway, thereby reducing inflammation and relieving insulin resistance. Therefore, based on effective control of blood glucose, NLRP3 inflammasome and its related inflammatory factors may become important targets for Tai Chi intervention to ameliorate pre-diabetes.

## Data availability statement

The original contributions presented in the study are included in the article/supplementary material. Further inquiries can be directed to the corresponding authors.

## Ethics statement

The studies involving human participants were reviewed and approved by Ethics Committee of Jishou University (approval number: JSDX-2021-0055). Written informed consent to participate in this study was provided by the participants’ legal guardian/next of kin.

## Author contributions

SH, PC, and XW designed the study. SH and XW drafted the manuscript. SH and YH drew the figures and filled the table. XW, PC, PLi and PLong revised the manuscript. All authors contributed to the article and approved the submitted version.

## Funding

This work was supported by grants from the Philosophy and Social Science Research Project of the Hubei Education Department (21Q050), the Key scientific research project of the Hunan Provincial Department of Education (20A414). The Central Government guides local funds for scientific and Technological Development (XZ202201YD0024C), Key R & D Program of Hubei Province (2021BGD010), Hubei Province Scientific and Technological Research Project (D20201306), Hubei Province Key Project of Research and Development Plan (to XW), Hubei Province Health Research Project (WJ2019-01), Hubei Medical Youth Tip-Top Talent (to XW), and Leading Talent Program of Yangtze Talent Project (to XW).

## Conflict of interest

The authors declare that the research was conducted in the absence of any commercial or financial relationships that could be construed as a potential conflict of interest.

## Publisher’s note

All claims expressed in this article are solely those of the authors and do not necessarily represent those of their affiliated organizations, or those of the publisher, the editors and the reviewers. Any product that may be evaluated in this article, or claim that may be made by its manufacturer, is not guaranteed or endorsed by the publisher.
